# Safe Resection of a Giant Mediastinal Liposarcoma with Severe Cardiac Compression

**DOI:** 10.70352/scrj.cr.25-0565

**Published:** 2026-03-07

**Authors:** Kazuhiro Mizota, Mikihiro Kohno, Fumihiko Kinoshita, Keigo Ozono, Tomoyoshi Takenaka, Tomoharu Yoshizumi

**Affiliations:** 1 Department of Surgery and Science, Graduate School of Medical Sciences, Kyushu University, Fukuoka, Fukuoka, Japan; 2 Department of Thoracic Surgery, Kyushu University Hospital, Fukuoka, Fukuoka, Japan

**Keywords:** oncologic emergency, liposarcoma, mediastinal tumor, mediastinal mass syndrome, extracorporeal membrane oxygenation (ECMO)

## Abstract

**INTRODUCTION:**

Dedifferentiated liposarcomas of the mediastinum are exceedingly rare, and surgical resection is the primary treatment of choice. Mediastinal mass syndrome (MMS) is an oncological emergency characterized by compression or invasion of the heart, great vessels, or trachea by a large mediastinal tumor, particularly during the induction of anesthesia. We report a case of a giant dedifferentiated liposarcoma in the anterior mediastinum, surgically resected due to severe cardiac compression and presenting as an oncological emergency.

**CASE PRESENTATION:**

A 70-year-old male presented with palpitations, generalized fatigue, and chest tightness. Chest radiography revealed an enlarged mediastinal shadow, prompting referral to our hospital. CT revealed a rapidly growing, giant anterior mediastinal tumor measuring 22 × 14.5 × 8.5 cm. The mass caused significant cardiac compression and extended into the pleural cavity. A CT-guided percutaneous biopsy confirmed a dedifferentiated liposarcoma. Given the patient’s presentation of tachycardia and relatively low blood pressure secondary to the large tumor, a semi-urgent surgical resection was planned. Surgical resection was performed with veno-arterial extracorporeal membrane oxygenation (VA-ECMO) on standby, anticipating potential hemodynamic decompensation during the induction of general anesthesia and surgery. Remarkably, the patient’s hemodynamics remained stable throughout the induction of general anesthesia, without requiring VA-ECMO support. A clamshell incision allowed for complete tumor resection, including a portion of the pericardium. Postoperatively, the patient recovered uneventfully, except for transient paroxysmal atrial fibrillation and heart failure. The final pathological diagnosis confirmed dedifferentiated liposarcoma, with tumor cells affecting the pericardial and anterior chest wall surgical margins. The patient received postoperative radiation therapy and adjuvant chemotherapy and has remained free of recurrence for 1 year after surgery.

**CONCLUSIONS:**

We successfully performed semi-urgent surgery in a patient with a rapidly growing, giant anterior mediastinal mass causing severe cardiac compression, with VA-ECMO on standby. As MMS constitutes an oncologic emergency, careful assessment of subjective symptoms and imaging findings is required to determine the need for preparing or initiating extracorporeal life support.

## Abbreviations

ECLS
: extracorporeal life support
ECMO
: extracorporeal membrane oxygenation
MMR
: mediastinal mass ratio
MMS
: mediastinal mass syndrome
MTR
: mediastinum-to-thorax ratio
VA-ECMO
: veno-arterial extracorporeal membrane oxygenation

## INTRODUCTION

Liposarcomas are malignant soft-tissue tumors that most commonly occur in the extremities, with primary mediastinal involvement in approximately 1% of cases.^1)^ Dedifferentiated liposarcoma has a poor prognosis, with a 5-year survival rate of approximately 30%.^2,3)^ Surgical resection is the mainstay of curative treatment. Mediastinal liposarcomas tend to grow to a large size, ranging from 2.2 to 61 cm in greatest dimension (median, 16 cm),^4)^ and can readily compress adjacent structures, including the heart, great vessels, and trachea. This clinical condition is referred to as MMS. MMS is an oncological emergency characterized by a high risk of hemodynamic decompensation, particularly during surgical procedures and the induction of general anesthesia, as a result of cardiovascular or airway compression. In such situations, ECLS may be required to prevent catastrophic circulatory collapse.

Here, we report a case of a giant dedifferentiated liposarcoma of the anterior mediastinum that caused severe cardiac compression in a patient who presented with preoperative tachycardia and hypotension, suggesting a high risk of MMS. The tumor was safely and successfully resected under standby ECMO support.

## CASE PRESENTATION

A 70-year-old male with a large anterior mediastinal tumor was referred to our institution. He reported worsening chest tightness in the supine position, palpitations, and general fatigue. On admission, his vital signs indicated sinus tachycardia (118 beats/min) and hypotension (81/58 mmHg).

**Fig. 1:**
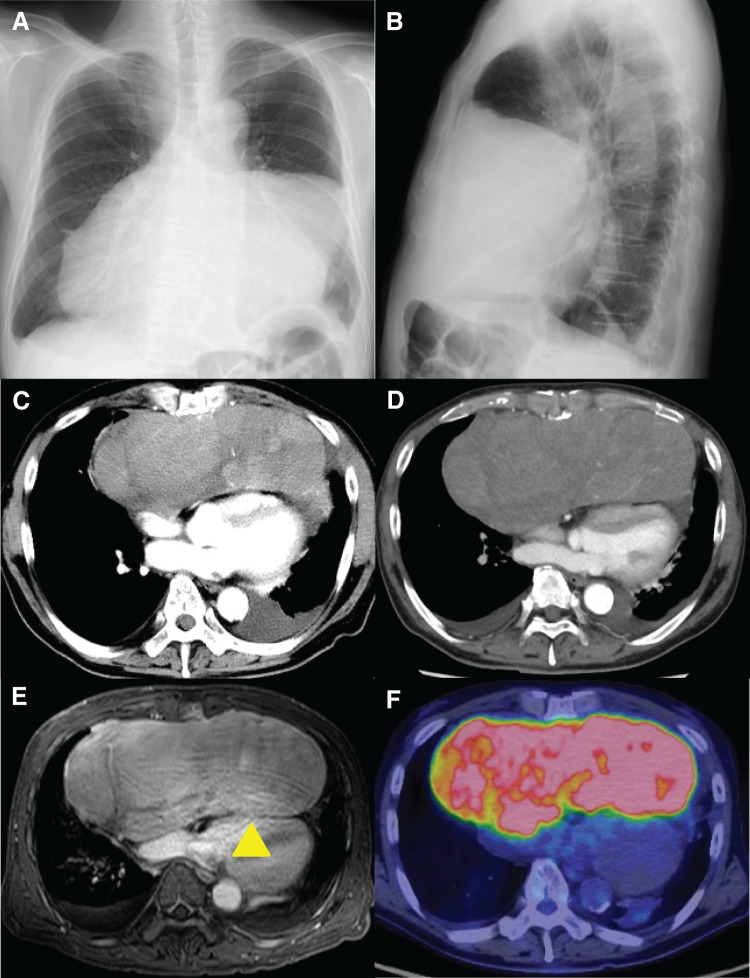
Chest X-ray shows enlarged anterior mediastinal shadow (**A**: posteroanterior view; **B**: lateral view). Chest contrast-enhanced CT (**C**: 39 days before the surgery; **D**: 4 days before the surgery) scan confirms a rapidly growing, giant lobulated and contrast-enhanced tumor of the anterior mediastinum, measuring 18 × 13 × 7 cm (**C**) with cardiac compression and extension into both pleural cavities. CT image 4 days before the surgery shows that the tumor presented a rapid growth to 22 × 14.5 × 8.5 cm (**D**). Contrast-enhanced magnetic resonance imaging suggests tumor invasion to the left anterior chest wall and pericardium close to the right atrium (**E**, arrowhead). PET-CT reveals increased uptake of fluorodeoxyglucose in the mass (maximum standardized uptake value = 21.5) (**F**).

Imaging studies confirmed the presence of a large anterior mediastinal mass (**Fig. 1A** and **1B**). Contrast-enhanced chest CT performed at the referring hospital 39 days before surgery demonstrated a huge, lobulated, contrast-enhancing tumor in the anterior mediastinum measuring 18 × 13 × 7 cm, with cardiac compression and extension into both pleural cavities (**Fig. 1C**). CT-guided percutaneous tumor biopsy performed after referral to our hospital suggested a dedifferentiated liposarcoma. A follow-up CT scan obtained 4 days before surgery showed rapid tumor growth to 22 × 14.5 × 8.5 cm (**Fig. 1D**). Contrast-enhanced MRI suggested tumor invasion of the left anterior chest wall and pericardium near the right atrium (**Fig. 1E**). PET-CT revealed increased fluorodeoxyglucose uptake in the mass (**Fig. 1F**), without evidence of distant metastasis. Echocardiography revealed significant compression of both the right and left ventricles by the mass, with an ejection fraction of 63.8% and no severe valvular disease.

**Fig. 2:**
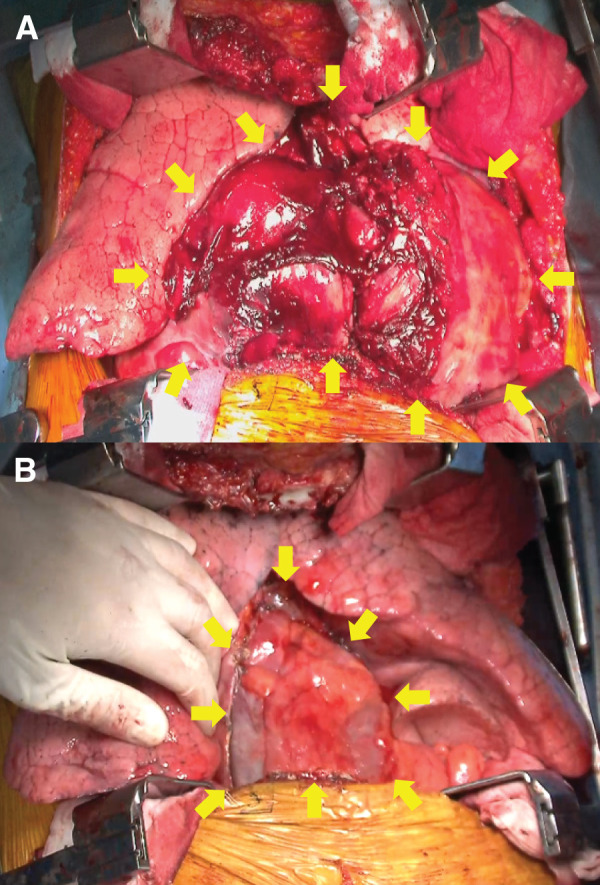
Intraoperative findings. A giant mass occupies both thoracic spaces (**A**) (yellow arrows indicate the tumor). Operative findings after tumor resection. The mass is resected along with the pericardium (**B**) (yellow arrows indicate the pericardial margin).

Given the rapid tumor growth, clinically assessed as progressive enlargement on serial imaging, together with its large size causing severe cardiac compression and tumor-related symptoms (tachycardia and hypotension), semi-urgent surgical resection was planned. Prior to surgery, a multidisciplinary conference was held with thoracic surgeons, anesthesiologists, cardiovascular surgeons, nurses, and perfusionists, during which we agreed on predefined criteria for initiating VA-ECMO in the event of hemodynamic decompensation (e.g., severe hypotension, hypoxemia, or cardiovascular collapse during anesthesia induction or surgery) and shared the specific procedural steps. In preparation for emergency ECMO cannulation, 18-gauge vascular access was secured in the right internal jugular vein, right femoral vein, and left femoral artery, enabling rapid ECMO cannula insertion and immediate initiation of ECMO in the event of hemodynamic collapse. However, the patient’s hemodynamics remained stable during anesthesia induction without the need for ECMO. A clamshell approach was chosen because the tumor extended into both pleural cavities, making adequate surgical exposure difficult to achieve with either a median sternotomy or a hemi-clamshell approach. In addition, given the massive size of the tumor and its severe cardiac compression, the clamshell incision provided wide bilateral exposure and allowed rapid relief of cardiac compression by elevating the tumor in the event of an emergency. The chest was accessed through the fourth intercostal space (**Fig. 2A**) and the tumor involved the left internal mammary artery and veins. Although there was no direct invasion of the lungs or great vessels, the pericardium was broadly involved and was resected along with the tumor (**Fig. 2B**). Tumor resection was completed macroscopically, and the pericardial defect was reconstructed using an expanded polytetrafluoroethylene patch. The operative duration was 367 min, and intraoperative blood loss was 1200 mL, requiring transfusion of 4 units of red blood cell concentrate. The patient recovered uneventfully, except for transient paroxysmal atrial fibrillation and heart failure, and was discharged on POD 22.

**Fig. 3:**
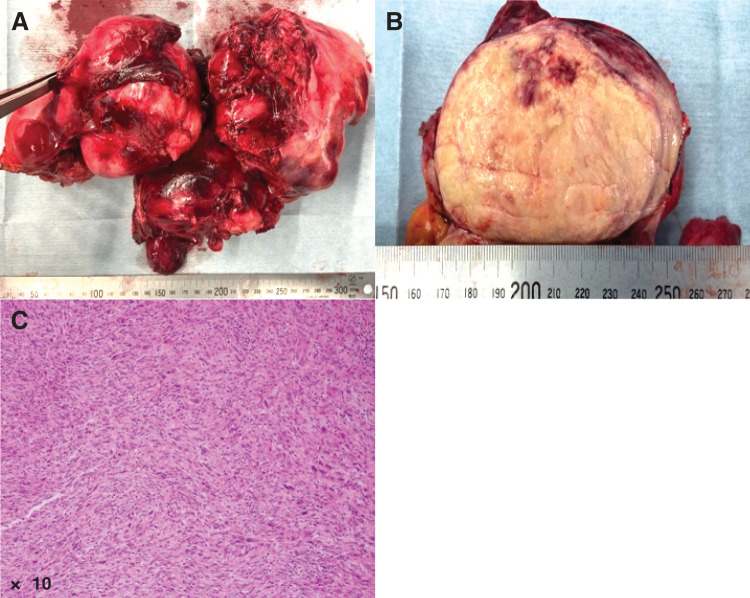
Macroscopic and histopathological findings. The mass in the anterior mediastinum is 24 × 14 × 8 cm in size (**A**) and the cut surface shows yellow content with focal hemorrhage (**B**). Hematoxylin–eosin staining shows a proliferation of spindle- to polygonal-shaped tumor cells (×10) (**C**).

**Fig. 4:**
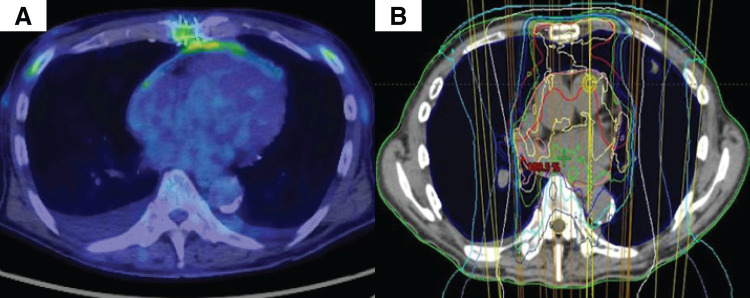
PET-CT at POD 46 shows no abnormal accumulation of fluorodeoxyglucose (**A**). Images showing postoperative radiation therapy planning (**B**).

The resected tumor measured 24 × 14 × 8 cm and weighed 1582 g (**Fig. 3A** and **3B**). Histopathological examination confirmed dedifferentiated liposarcoma (**Fig. 3C**). The resection margins of the pericardium and anterior chest wall were positive for tumor cells, indicating an R1 resection. A PET-CT scan 1 month after discharge revealed no evidence of recurrence or residual tumor (**Fig. 4A**). Because pathological evaluation revealed an R1 resection, the patient received postoperative radiation therapy (66 Gy in 33 fractions; **Fig. 4B**) after multidisciplinary discussion with medical oncologists and radiation oncologists. Subsequently, chemotherapy was administered because the tumor was extremely large and therefore considered to confer a high risk of recurrence. The chemotherapy regimen (doxorubicin 80 mg/m^2^ [60% dose] over 72 h, combined with ifosfamide 0.8 g/m^2^ [80% dose]) was selected in accordance with the clinical practice guidelines on the management of soft tissue tumors (2020).^5)^ The patient remained recurrence-free for 1 year after surgery.

## DISCUSSION

According to the 2020 World Health Organization classification of tumors, liposarcomas are malignant adipocytic tumors classified into 5 subtypes: 1) well-differentiated liposarcoma, 2) dedifferentiated liposarcoma, 3) myxoid liposarcoma, 4) pleomorphic liposarcoma, and 5) myxoid pleomorphic liposarcoma. Because dedifferentiated liposarcomas respond poorly to chemotherapy and radiotherapy, surgical resection is the treatment of choice. Nevertheless, it is associated with high rates of local recurrence and distant metastasis, with a poor prognosis and a 5-year survival rate of approximately 30%.^3,6)^

Furthermore, mediastinal tumors are often asymptomatic or take a long time to produce symptoms, leading to their discovery at an advanced size. Due to its limited space within the mediastinum, it is particularly susceptible to compression and invasion of surrounding organs as the tumor enlarges. Consequently, compression of the surrounding organs, such as the heart, great vessels, and trachea, can cause adverse cardiovascular and respiratory events. Circulatory and respiratory collapse caused by a large mediastinal tumor in an anesthetized patient is referred to as MMS. The main causes include worsening tumor compression due to muscle relaxation, increased intrathoracic pressure, reduced thoracic cavity size from positive-pressure ventilation, and diaphragmatic elevation.^7,8)^ The incidence of MMS associated with mediastinal liposarcoma has not been clearly established. MMS has been more frequently described in association with malignant lymphoma, germ cell tumors, thymic tumors, and large intrathoracic goiters.^8)^ A common feature of these tumors is their origin in the anterior mediastinum and their tendency for rapid growth, which predisposes patients to cardiopulmonary compromise. Among patients with tumors ≥130 cm^3^, perioperative complications associated with MMS, including atrial fibrillation, hypoxemia, respiratory distress, and airway edema, were reported in 3.8% preoperatively and 10.5% postoperatively.^9)^ Preoperative risk assessment for MMS includes: 1) clinical findings—airway symptoms in the supine position and a history of syncope or arrhythmia; 2) physiological tests—pulmonary function tests showing a ≥50% reduction in peak expiratory flow and echocardiographic abnormalities; and 3) imaging findings—airway narrowing ≥50%, MTR ≥50%, and MMR ≥56%.^10,11)^ The MTR was defined as the ratio of the mediastinal tumor size to the thoracic transverse diameter measured on chest radiography, whereas the MMR was defined as the ratio of the maximum tumor diameter to the thoracic transverse diameter measured on CT. In the present case, the MTR was 82.5% and the MMR was 79.1%, classifying it as a high-risk for MMS (**Supplementary Fig. 1**).

An oncological emergency refers to various urgent conditions caused by malignant tumors. These conditions include metabolic, cardiac, neurological, respiratory, and gastrointestinal disorders and may arise at any stage of malignancy. Many of these emergencies are life-threatening and require prompt intervention.^12)^

Although many cases of large mediastinal tumors have been reported, this case is notable for its rapid growth and pronounced cardiac compression. CT revealed a 4-cm increase in tumor diameter over approximately 1 month, leading to chest symptoms due to cardiac compression. In this case, the patient exhibited tachycardia and hypotension, indicating imminent respiratory and circulatory collapse, and qualifying as an oncological emergency. After careful preoperative assessment of MMS risk using both clinical and imaging findings, peripheral VA-ECMO was prepared for induction of general anesthesia. Fortunately, the patient's respiratory and circulatory status remained stable during anesthesia induction without ECMO, despite muscle relaxant administration, initiation of positive-pressure ventilation, and positional changes. No significant intraoperative complications or hemodynamic fluctuations were observed from incision to closure, despite the complexity of tumor resection and pericardial reconstruction. However, in cases of severe cardiac compression presenting as an oncological emergency, having ECMO on standby is crucial.

## CONCLUSIONS

We successfully performed semi-urgent surgery in a patient with a rapidly growing, giant anterior mediastinal liposarcoma causing severe cardiac compression, with VA-ECMO on standby. As MMS constitutes an oncological emergency, careful assessment of symptoms and imaging findings is essential to determine the need for initiating ECMO.

## SUPPLEMENTARY MATERIALS

Supplementary Fig. 1 Measurement of the MTR on chest radiography and MMR on CT. (A) The MTR was calculated as the ratio of the transverse diameter of the mediastinal tumor (a) to the thoracic transverse diameter (b). (B) The MMR was calculated as the ratio of the maximum tumor diameter (c) to the thoracic transverse diameter (d).

## DECLARATIONS

### Availability of data and materials

The raw data were generated by the Department of Surgery and Science at the Graduate School of Medical Sciences, Kyushu University, Fukuoka, Japan. The data supporting the findings of this study are available from the corresponding author, M. Kohno, upon request.
