# Supplementary figures and images for: Safe Resection of a Giant Mediastinal Liposarcoma with Severe Cardiac Compression
Source: Surg Case Rep. 2026 Mar 7;12(1):25-0565. doi: 10.70352/scrj.cr.25-0565 (PMC12975342; doi:10.70352/scrj.cr.25-0565)

# Supplementary Fig. 1

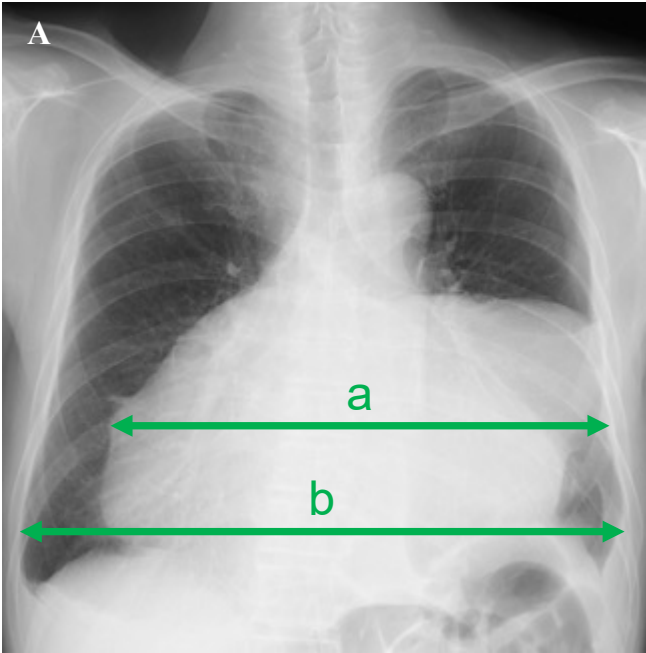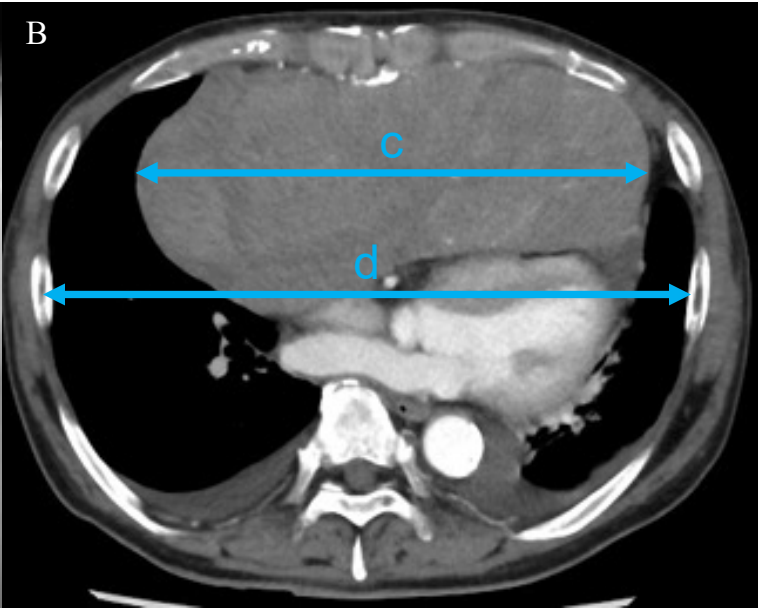

Supplement: Supplementary Fig. 1 — Measurement of the MTR on chest radiography and MMR on CT. (A) The MTR was calculated as the ratio of the transverse diameter of the mediastinal tumor (a) to the thoracic transverse diameter (b). (B) The MMR was calculated as the ratio of the maximum tumor diameter (c) to the thoracic transverse diameter (d). [file scr-12-01-25-0565-s001.pdf]
